# Role of Factors in Embryo Implantation and Placental Development

**DOI:** 10.3390/biomedicines14071639

**Published:** 2026-07-21

**Authors:** Micol Massimiani

**Affiliations:** Departmental Faculty of Medicine, Saint Camillus International University of Health Sciences, Via di Sant’Alessandro 8, 00131 Rome, Italy; micol.massimiani@unicamillus.org

## 1. Introduction

Embryo implantation and placental development are fundamental processes for the establishment and maintenance of a successful pregnancy. Implantation represents a critical and highly regulated event in early gestation, requiring precise synchronization between a competent blastocyst and a receptive endometrium. This complex process is orchestrated by a wide range of molecular factors, signaling pathways, and cellular interactions that coordinate embryo–maternal communication [1,2]. Disruptions in this finely tuned crosstalk may result in implantation failure, recurrent pregnancy loss, and infertility, highlighting the need for a deeper understanding of the mechanisms governing early pregnancy establishment [3].

Following successful implantation, the rapid growth and increasing metabolic demands of the developing embryo necessitate the formation of an efficient maternal–fetal interface. This role is fulfilled by the placenta, a transient yet essential organ that mediates nutrient and gas exchange, regulates immune tolerance, and produces hormones critical for pregnancy progression. Placental development is a dynamic process involving trophoblast differentiation, invasion, vascular remodeling, and the establishment of uteroplacental circulation. These tightly coordinated events are finely regulated by a complex network of molecular pathways, growth factors, cytokines, hormones, and cell–cell interactions that ensure proper placental formation and function throughout pregnancy [1,4]. Aberrant placentation has been associated with several pregnancy complications, including preeclampsia, fetal growth restriction, preterm birth, and long-term adverse health outcomes for both the mother and offspring [1,5].

Over recent decades, considerable advances have been made in elucidating the molecular and cellular mechanisms underlying embryo implantation and placental development. Nevertheless, many aspects of the regulatory networks involved remain incompletely understood. Increasing evidence points to the pivotal role of growth factors, cytokines, hormones, extracellular vesicles, non-coding RNAs, epigenetic regulators, and immune mediators in coordinating embryo–endometrium interactions and placental formation. Understanding how these factors contribute to physiological pregnancy and how their dysregulation leads to reproductive and obstetric disorders may facilitate the identification of novel diagnostic biomarkers and therapeutic targets [6,7,8,9,10].

The purpose of this Special Issue, “Role of Factors in Embryo Implantation and Placental Development”, is to gather original research articles and comprehensive reviews that provide novel insights into the factors and molecular pathways regulating blastocyst–endometrium crosstalk, implantation, trophoblast function, and placental development. The contributions in this collection explore diverse aspects of reproductive biology and placental physiology, highlighting emerging mechanisms involved in pregnancy establishment and maintenance, as well as potential therapeutic strategies for infertility and pregnancy-related complications.

Across these contributions, a multifaceted picture emerges, emphasizing the complexity of embryo–maternal interactions and placental biology. The growing understanding of the molecular determinants governing implantation and placentation offers promising opportunities for improving reproductive outcomes and developing innovative approaches for the prevention, diagnosis, and treatment of pregnancy disorders. This Editorial briefly summarizes the articles included in this Special Issue and invites readers to explore the valuable insights they provide into these essential processes of human reproduction.

## 2. An Overview of the Published Articles

The articles in this collection provide complementary perspectives on the molecular, cellular, and clinical factors involved in embryo implantation and placental development. Together, they highlight the complexity of embryo–maternal interactions and underscore the importance of identifying regulatory mechanisms that may serve as biomarkers or therapeutic targets in reproductive disorders.

The first group of studies focuses on the early stages of pregnancy establishment, examining molecular and hormonal factors that regulate endometrial receptivity and embryo implantation. These contributions highlight the importance of the inflammatory and endocrine microenvironment in determining implantation success. Ardizzone et al. [11] investigated the role of Pentraxin 3 (PTX3) in follicular fluid from women undergoing in vitro fertilization and identified it as a strong predictor of implantation failure and follicular inflammation, outperforming classical inflammatory mediators such as TNF-α and IL-1β. These results suggest that PTX3 may represent a promising biomarker for predicting ART outcomes and improving patient stratification. Complementing these findings, Yusuf et al. [12] explored the effects of supraphysiological testosterone exposure on endometrial receptivity in a rat model. The authors demonstrated that testosterone administration impairs uterine morphology and dysregulates key components of the leukemia inhibitory factor (LIF) signaling pathway, leading to reduced expression of LIF, LIFR, JAK1, and STAT3, alongside increased MUC1 expression. Their study provides further evidence of the critical role of hormonal balance in maintaining a receptive endometrium and supporting embryo implantation.

The remaining contributions shift the focus from implantation to placental development and the molecular mechanisms underlying placental function and pregnancy complications. Together, they provide insights into placental morphogenesis, genetic susceptibility to obstetric disorders, and potential therapeutic approaches. A comprehensive overview of placental biology is provided by Vornic et al. [13], who review the morphological and molecular events underlying placental development and function. The authors discuss the coordinated roles of trophoblast invasion, spiral artery remodeling, and chorionic villi formation, emphasizing the contribution of cytokines, growth factors, and signaling pathways, including VEGF and Notch signaling, in establishing a functional maternal–fetal interface. Their work integrates current knowledge on placental morphogenesis and offers valuable insights into the mechanisms supporting successful pregnancy.

The relationship between molecular determinants and pregnancy complications is addressed by Kurmanova et al. [14], who evaluated thrombophilia-associated genetic polymorphisms in women with reproductive disorders, including fetal loss syndrome, postpartum hemorrhage, and preeclampsia. Their analysis revealed distinct genetic profiles associated with specific obstetric phenotypes, identifying polymorphisms in F13, ITGA2, MTR, and MTHFR genes as potential contributors to disease susceptibility and clinical outcomes. These findings reinforce the importance of personalized approaches in the assessment and management of pregnancy-related disorders.

Finally, Salvi et al. [15] investigated the effects of pravastatin on placental signaling pathways in chorionic villous explants from women with preeclampsia. The study identified Epidermal Growth Factor-Like Domain 7 (EGFL7) as a novel target modulated by pravastatin and revealed heterogeneous responses among patients, distinguishing high responders from low responders according to the activation of EGFL7 and Notch signaling pathways. These findings not only provide new insights into the molecular mechanisms underlying pravastatin action but also support the development of personalized preventive and therapeutic strategies for preeclampsia.

Collectively, these contributions expand our understanding of the factors governing embryo implantation and placental development, from physiological regulatory mechanisms to pathological alterations associated with infertility and pregnancy complications. They further highlight the potential of molecular biomarkers and targeted interventions to improve reproductive outcomes and maternal–fetal health.

## 3. Conclusions

The studies collected in this Special Issue provide complementary insights into the molecular, cellular, and genetic mechanisms regulating embryo implantation and placental development. Together, they advance current understanding of embryo–maternal communication, endometrial receptivity, trophoblast function, and placental maturation, while highlighting emerging biomarkers and molecular pathways with potential clinical relevance for reproductive and pregnancy-related disorders.

Despite these advances, further experimental, translational, and clinical studies are needed to validate these findings, clarify the biological significance of emerging molecular markers, and facilitate their translation into personalized approaches for reproductive medicine. Integrating basic research with clinical investigation will be essential to improve the prediction, prevention, and management of conditions such as implantation failure, recurrent pregnancy loss, preeclampsia, and other placental disorders, ultimately contributing to better maternal and fetal health outcomes.

We sincerely thank all authors for their valuable contributions and the reviewers for their expertise and dedication throughout the peer-review process. We hope that the articles presented in this collection will stimulate further research into the mechanisms governing embryo implantation and placental development, ultimately fostering innovative strategies to improve reproductive success and pregnancy health.
